# Understanding and Targeting the Wnt/**β**-Catenin Signaling Pathway in Chronic Leukemia

**DOI:** 10.4061/2011/329572

**Published:** 2011-12-04

**Authors:** S. Thanendrarajan, Y. Kim, I. G. H. Schmidt-Wolf

**Affiliations:** Department of Internal Medicine III (Hematology and Oncology), Center for Integrated Oncology (CIO), University of Bonn, Sigmund-Freud Stra*β*e 25, 53127 Bonn, Germany

## Abstract

It has been revealed that the Wnt/**β**-catenin signaling pathway plays an important role in the development of solid tumors and hematological malignancies, particularly in B-cell neoplasia and leukemia. In the last decade there have been made experimental approaches targeting the Wnt pathway in chronic leukemia. In this paper we provide an overview about the current state of knowledge regarding the Wnt/**β**-catenin signaling pathway in chronic leukemia with special focus on therapeutic options and strategies.

## 1. Introduction

### 1.1. Treatment of Chronic Leukemia

Chronic myeloid leukemia (CML) is a one of the myeloproliferative diseases and represents 15–20% of all adult leukemia types. Depending on clinical aspects and laboratory findings, CML can be divided in three phases. Typically CML begins in the chronic phase, and over several years it progresses to the accelerated phase and finally to the blast crisis. By the introduction of the bcr-abl tyrosine kinase inhibitor (TKI) imatinib and the second-generation TKIs dasatinib and nilotinib, the treatment of CML has been revolutionized. The overall survival rate of patients with CML has been clearly improved. Nevertheless, resistance and incompatibilities against TKIs are well known, and a molecular remission is difficult to achieve [1]. Curing patients with CML could only be achieved by performing allogeneic stem cell transplantation (ASCT).

Chronic lymphocytic leukemia (CLL) belonging to the indolent B-non-Hodgkin lymphoma (B-NHL) is the most common leukemia in the adult. The treatment of CLL mainly depends on the clinical stage of the disease, which is determined by the Binet staging system (A–C). In the early stage a “watch and wait” strategy is recommended. In progress of CLL, various chemotherapeutical options are determined. The application of fludarabine, cyclophosphamide, and rituximab (FCR) is one of the treatment standards in the first-line therapy of CLL [2]. Further alternative therapeutic options, particularly in progress or relapse and under consideration of several clinical aspects, have been established. These include the application of bendamustin and rituximab (R-Benda), chlorambucil, and alemtuzumab [2]. Despite the progress made in developing effective chemotherapeutic regimes, CLL remains an incurable disease. The only curative approach in CLL represents the performance of ASCT.

### 1.2. Wnt/*β*-Catenin Signaling Pathway

The Wingless-Int (Wnt) signaling pathway plays an essential role in embryogenesis and proliferation, survival, and differentiation of hematopoietic stem cells (HSCs) [3, 4]. It represents a complex network with mechanisms of self-regulation through positive and negative feedback [5]. There have been described a large number of Wnt proteins that activate various pathways in cells categorized as canonical and noncanonical Wnt pathways [6]. In this paper primarily the Wnt/*β*-catenin signaling pathway is focused. Influencing the expression of genes involved in cell proliferation, differentiation and apoptosis are primarily due to the activation of the canonical *β*-catenin and lymphoid enhancer factor (LEF)/T-cell factor (TCF) pathway [7]. Wnt proteins mainly stabilize cytoplasmic *β*-catenin that has two essential functions: it is a very important element of many intracellular signaling pathways, including the Wnt pathway, but it also takes part in creating intercellular adhesive junctions [4]. Signaling through *β*-catenin is mainly regulated by modulating its degradation and nuclear translocation [8].

**Figure 1:**
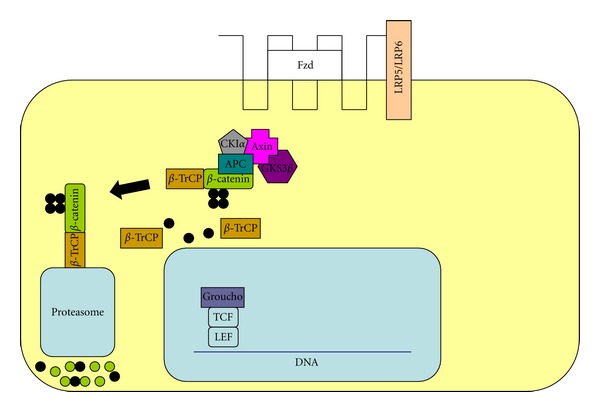
The inactivated Wnt pathway. Fzd: frizzled receptor, LPR5/LPR5: lipoprotein receptor-related protein 5/6, CKI*α*: casein kinase I alpha; GSK3*β*: glycogen synthase kinase 3 beta, *β*-TrCP: *β*-transducin repeat-containing protein, DNA: deoxyribonucleic acid, TCF: T-cell factor, and LEF: lymphoid enhancer factor.

In most normal cells the Wnt pathway is inactive. In the cytosol a number of proteins such as adenomatous polyposis coli (APC), axin, glycogen synthase kinase 3 (GSK3), casein kinase alpha (CK1*α*), and *β*-catenin form the destruction complex [8, 9]. In the absence of an activating signal, phosphorylation of *β*-catenin by GSK3 acting in conjunction with APC and axin causes *β*-catenin to interact with the ligase *β*-transducin repeat-containing protein (*β*-TrCP) which results in its ubiquitination and degradation, executed by proteasome [8]. Thereby, a low level of cellular *β*-catenin is achieved in the nucleus. Here, the transcription factor of the LEF/TCF family, together with other proteins such as groucho, binds to DNA and inhibits gene expression as a transcriptional repressor (Figure 1).

**Figure 2:**
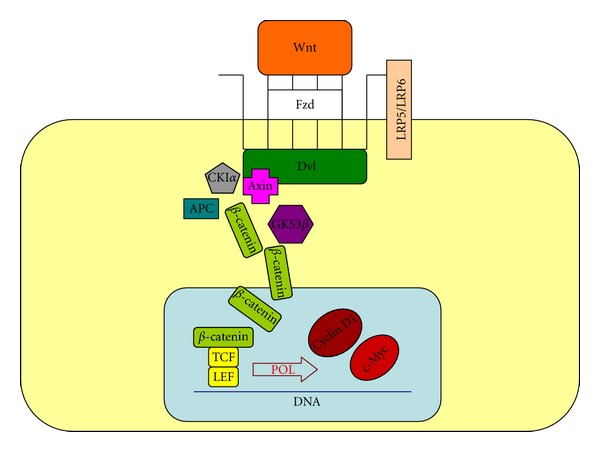
The activated Wnt pathway. Wnt: wingless-int; POL: ribonucleic acid polymerase; Dvl: disheveled.

Activation of the signal transduction pathway is performed by Wnt proteins. These are secreted glycoproteins that act as ligands for membrane receptors belonging to the frizzled (Fzd) family of proteins and their coreceptors lipoprotein receptor-related protein 5 and 6 (LRP 5 and 6) [10]. Consequently the protein disheveled (Dvl) is activated and induces the dissociation of the destructive complex. Thereby, activity of GSK3 is inhibited, and an increase of free pool *β*-catenin with its stabilization and translocation into the nucleus is developed [8, 11]. In the nucleus *β*-catenin acts as a transcriptional coactivator for the LEF/TCF family of transcription factors [12]. In the final step of the *β*-catenin-LEF/TCF signaling pathway, nuclear *β*-catenin binds pontin52-TATA-binding protein and displaces groucho-related gene or CREB-binding protein corepressors from LEF/TCF resulting in stimulation of transcription of important growth regulatory genes, including cyclin D1 and c-Myc [8, 13] (Figure 2).

There have been defined many other agents that work as physiological promoters of the Wnt pathway, such as dishevelled-associated kinase (DAK), casein kinase I epsilon (CK I*ε*), casein kinase II (CK II), integrin-linked kinase (ILK), B-cell lymphoma 9 (BCL9) and BCL9-2/BCL9-like (B9L), together with pygopus [8, 10, 14–16]. These agents are involved in different parts of the Wnt/*β*-catenin pathway and lead to an increased accumulation of cellular *β*-catenin with augmented assembly of Wnt target genes. Axin1, axin2, HMG-box transcriptional factor (HBP1), Dapper1 (Dpr1), Chibby, and inhibitor of *β*-catenin and TCF (ICAT), on the contrary, represent complex negative regulators of the Wntsignaling pathway. They antagonize the Wnt pathway by decreasing the cellular *β*-catenin, disrupting the *β*-catenin/LEF-1 complexes, and repressing the Wnt target genes [7, 13, 17–19].

## 2. Wnt Signaling in Cancer and Chronic Leukemia

There is strong evidence that defects in the Wnt pathway are involved in the development of several types of solid tumors, for example, colorectal, prostate, and breast cancer [8, 20–22]. It has been known for a long time that hematological malignancies, such as chronic myeloid and lymphocytic leukemia, mantle cell lymphoma, multiple myeloma, and acute myeloid leukemia may occur partly because of the constitutive activation of Wnt/*β*-catenin canonical signaling pathway [23, 24]. Especially mutations in downstream components of the Wnt pathway such as APC, axin, or *β*-catenin have been found to be responsible for the genesis of human cancers due to aberrant signaling activity [9, 25]. Genetic defects in APC are responsible for a heritable predisposition to colon cancer. APC protein binds *β*-catenin, retains it in the cytoplasm, and facilitates the proteolytic degradation of *β*-catenin. Abrogation of this negative regulation allows *β*-catenin to translocate to the nucleus and to form a transcriptional activator complex with the DNA-binding protein LEF-1 [26]. Expression of mutant oncogenic forms of *β*-catenin identified in cancer cells resulted in higher levels of transcriptional activity. The results suggest that a cancer pathway driven by Wnt proteins, or mutant forms of *β*-catenin, may involve the formation of a persistent transcriptionally active complex of *β*-catenin and LEF-1 [11].

The treatment of the Philadelphia chromosome-positive CML is highly effective due the inhibition of bcr-abl kinase activity by the TK inhibitor imatinib. Nevertheless, it is difficult to achieve molecular remission, suggesting that leukemia stem cells (LSCs) remain in the patient. In vivo after imatinib treatment, LSCs not only remained but also accumulated increasingly in bone marrow of CML mice [1]. In this context it is discussed that the Wnt pathway plays a vital role in the survival and self-renewal of LSC [1]. Zhao et al. revealed that *β*-catenin deletion causes a reduction in the ability of mice to develop bcr-abl-induced CML [27]. Intriguingly, genetic analysis of transformed blasts from patients in blast crisis has identified numerous members of the Wnt/*β*-catenin pathway as being activated. Increased activity of these pathways correlates with poor response and eventual disease progression. In addition to these data, evidence is emerging associating survival of blast cell with Wnt activity, leading to the hope that Wnt inhibitors will increase the likelihood of eradicating these cells [28]. A comparison of the gene signatures of chronic, accelerated, and blast phases suggests that the progression of chronic phase to advanced phase CML (accelerated phase and blast crisis) is a two-step process, with new gene expression changes occurring early in accelerated phase before the accumulation of increased numbers of leukemia blast cells. In this process deregulation of the Wnt/*β*-catenin pathway seems to represent an important aspect [29].

A significant role in the development of CLL is attributed to the Wnt pathway. It has been demonstrated that the Wnt signaling pathway is activated in CLL cells and that uncontrolled Wnt/*β*-catenin signaling may contribute to the defect in apoptosis that characterizes this malignancy [30]. It is thought to be responsible for the extended survival of CLL cells in vivo [31]. In primary CLL cells, Wnt proteins are overexpressed, and physiological inhibitors of this pathway are inactivated [32]. After upregulation of *β*-catenin due to Wnt stimulation, it cooperates with the transcription factor LEF-1, which is overly expressed in CLL by more than three-thousand-fold compared to normal B cells [32]. Furthermore LEF-1 is regarded as an essential regulator of pathophysiological, relevant genes in CLL and several Wnt/*β*-catenin signaling components which fundamentally influence CLL cell survival [14, 33]. Similar findings have been made by Gutierrez et al. in analyzing the Wnt pathway by using gene expression profiling. As a result aberrant regulation of Wnt pathway target genes, ligands, and signaling members could be identified. Here, especially the constitutive Wnt pathway activation and the aberrant protein expression of LEF-1 in CLL was remarkable [33]. Even in patients with monoclonal B-cell lymphocytosis that is regarded as a premalignant condition of CLL, an expression of LEF-1 in CD19+/CD5+ cells could be identified [33]. It is assumed that LEF-1 plays an essential role in the leukemogenesis of CLL [33].

It has been revealed that certain Wnt and Wnt network target genes are expressed at higher or lower levels in CLL compared with normal B cells [34]. This includes upregulation of nuclear complex genes, as well as genes for cytoplasmatic proteins and Wnt ligands and their related receptors [34]. There has been identified epigenetic silencing of several negative regulators of the Wnt/*β*-catenin pathway. The balance between epigenetic downregulation of negative effector genes and increased expression of positive effector genes plays an outstanding role in the pathogenesis of CLL [34]. Especially the epigenetic downregulation of Wnt antagonists, such as Dickkopf (DKK) and WIF1 by hypermethylation, is one mechanism, perhaps the main mechanism that is permissive to active Wnt signaling in CLL [25, 34].

Furthermore, the overexpression of positive effectors in the Wnt pathway is also involved in the pathogenesis of CLL. In this model, DNA methylation, histone modifications, and altered expression of microRNA molecules interact to allow continuous Wnt signaling. All these mechanisms result in a common consequence, the activation of LEF-1/TCF transcription factors and subsequent target gene expression [25].

A large number of Wnt proteins such as Wnt3, Wnt5b, Wnt6, and Wnt10a, as well as the Wnt receptor Fzd3, are highly expressed in CLL, compared with normal B cells. Furthermore the Wnt/*β*-catenin-regulated transcription factor LEF-1 and its downstream target cyclin D1, are overexpressed in CLL. Lu et al. were able to demonstrate that a pharmacological inhibitor of GSK3*β*, SB-216763, activated *β*-catenin-mediated transcription and enhanced the survival of CLL lymphocytes [35]. Taken together, these results indicate that Wnt signaling genes are overexpressed and active in CLL. Uncontrolled Wnt signaling may contribute to the defect in apoptosis that characterizes this malignancy [35].

Dickkopf1 (DKK1) is known to antagonize Wnt signaling by direct high-affinity binding to the extracellular domain of the Wnt coreceptor LRP6. In B cells from patients with CLL, added DKK1 did not inactivate the Wnt pathway. The reason for this could be the absence of the binding domain of LRP6. It is estimated that in CLL cells every 6th LRP6 receptor is lacking the extracellular domain. Normal B cells proved to have significantly higher levels of extracellular, DKK1-binding domain of LRP6. On the other hand, a truncated LRP6 without extracellular DKK1-binding domain could lead to an uncontrollable activation of Wnt signaling in CLL [31].

## 3. Targeting the Wnt/***β***-Catenin Signaling Pathway

Cancer stem cells (CSCs) play a significant role in the development and recurrence of several cancers. At the same time Wnt/*β*-catenin signaling is important for the proliferation of CSCs. Therefore, inhibition of Wnt/*β*-catenin signaling is a promising treatment approach [36]. While substantial progress has been made in developing therapeutics targeting in other signal pathways, the Wnt pathway has remained an elusive therapeutic target [37]. However, in the recent years specific inhibitors of this pathway have been keenly researched and developed [36]. The variety of possible underlying mechanisms leading to *β*-catenin/LEF-1/TCF activation offers multiple options to target the aberrantly activated pathway in order to prevent target gene expression or their gene products to exert their tumorigenic function [25].

Over the last decade, numerous approaches have been developed to target the Wnt/*β*-catenin pathway in tumor cells, from antagonizing Wnt ligand secretion or binding to promoting *β*-catenin degradation to specifically blocking *β*-catenin-mediated transcriptional activity [20]. The binding of secreted Wnt ligands to their receptors offers an attractive and accessible target for therapeutic regulation of these signaling pathways. Wnt proteins, Wnt receptors, and secreted Wnt inhibitors are attractive as potential therapeutic agents and targets due to their extracellular location. Wnt signaling results in a diverse array of downstream intracellular events, many of which are not fully understood. The targeting of this pathway at the most upstream site of pathway activation also provides a strategic advantage for therapy [38]. There have been done promising researches and efforts on targeting the Wnt pathway in solid tumors and hematology malignancies, for example, colorectal, breast, prostate cancer, multiple myeloma, and acute myeloid leukemia [21, 22, 39–41].

Little is known about targeting the Wnt pathway in CML. Based on current knowledge, there have not been made breakthrough advances targeting the Wnt pathway in CML. However, the fact remains that *β*-catenin in the Wnt signaling pathway is essential for survival and self-renewal of LSCs in CML, providing a new strategy for targeting these cells [1]. Particularly in patients with progress, relapse, or incompatibilities under the treatment with TK inhibitors further therapeutic developments have to be made. Here, the Wnt pathway could play a fundamental role.

In contrast, recent researches indicate that the Wnt pathway is an attractive candidate for developing targeted therapies for CLL [30]. As one of the first, Lu et al. successfully targeted the Wnt pathway in CLL lymphocytes by using R-etodolac, an analog of a nonsteroidal anti-inflammatory drug, which diminished the Wnt/*β*-catenin signaling [35].

Furthermore Lu et al. identified the diuretic agent ethacrynic acid (EA) as a Wnt inhibitor. In vitro assays confirmed the inhibitory effect of EA on Wnt/*β*-catenin signaling. Cell viability assays showed that EA selectively induced cell death in primary CLL cells. Exposure of CLL cells to EA decreased the expression of Wnt/*β*-catenin target genes, including LEF-1, cyclin D1, and fibronectin. Immune coprecipitation experiments revealed that EA could directly bind to LEF-1 protein and destabilize the LEF-1/*β*-catenin complex [30]. Similar findings have been made by Jin et al.: a series of amides of ethacrynic acid were prepared and evaluated for their ability to inhibit Wnt signaling and decrease the survival of CLL cells. Reduction of the alpha-, beta-unsaturated carbon-carbon double bond of EA abrogated both the inhibition of Wnt signaling as well as the decrease in CLL survival. Preliminary mechanism of action studies suggests that these derivatives covalently modify sulfhydryl groups present on transcription factors important for Wnt/*β*-catenin signaling [42] (Table 1).

Another approach is the inhibition of the Wnt pathway cascade further upwards by using salinomycin [43]. Salinomycin is an antibiotic potassium ionophore with the ability to inhibit the development of breast cancer stem cells [43]. In Wnt-transfected HEK293 cells, salinomycin blocked the phosphorylation of the Wnt coreceptor LRP6 and induced its degradation. Ingrain, another potassium ionophore with activity against CSCs, revealed similar effects. In CLL cells with constitutive Wnt activation, nanomolar concentrations of salinomycin decreased the expression of Wnt target genes such as LEF-1, cyclin D1, and fibronectin, depressed LRP6 levels, and limited cell survival. Normal human peripheral blood lymphocytes resisted salinomycin toxicity. These results indicate that ionic changes induced by salinomycin and related drugs inhibit proximal Wnt signaling by interfering with LPR6 phosphorylation and thus impair the survival of cells that depend on Wnt signaling at the plasma membrane [43].

Nitric oxide-donating acetylsalicylic acid (NO-ASA) has been shown to possess an antineoplastic effect in Wnt/*β*-catenin active cancers. The effect of the paraisomer of NO-ASA on CLL cell survival in vitro and in a CLL-like xenograft mouse model was analyzed by Razavi et al. [44]. Apoptosis in primary CLL cells was determined, and interference of NO-ASA with Wnt/*β*-catenin signaling was analyzed through immunoblots of different pathway members. CLL-like JVM3 cells were subcutaneously inoculated into irradiated nude mice that were treated with 100 mg of *para*-NO-ASA/kg of body weight p.o. for 21 days. *para*-NO-ASA induced apoptosis in CLL cells, whereas healthy blood cells were not affected. In addition, cleavage of *β*-catenin and downregulation of *β*-catenin/LEF-1 targets were observed. *para*-NO-ASA exhibited strong antitumor efficacy in the xenograft mouse model with a tumor inhibition rate of 83.4%. *para*-NO-ASA selectively induces apoptosis in primary CLL cells and efficiently reduces tumor growth in a CLL-like xenograft model. As NO-ASA is orally available and is generally well tolerated, it might be a promising novel agent for CLL therapy [44].

Gandhirajan et al. used two small molecule inhibitors of the Wnt/*β*-catenin/LEF-1 pathway (CGP049090 and PKF115-584) in vitro and in vivo studies in order to antagonize LEF-1 [45]. By using nucleofection, small interfering RNA- (siRNA-) mediated knockdown of LEF-1 in primary CLL cells was performed. Then the LC50 of the two small molecules was evaluated using ATP-based cell viability assay. Inhibition of LEF-1 by siRNA leads to increased apoptosis of CLL cells and inhibited proliferation of JVM-3 cell lines (subcutaneous xenograft model) [45]. The two small molecule inhibitors efficiently killed CLL cells, whereas normal B cells were not significantly affected. Coimmunoprecipitation showed a selective disruption of *β*-catenin/LEF-1 interaction. In vivo studies exhibited tumor inhibition of 69% with CGP049090 and 57% with PKF115-584 [45]. Targeting LEF-1 might be a new and selective therapeutic approach in CLL.

## 4. Conclusion

The Wnt/*β*-catenin signaling pathway plays an outstanding role in the development of chronic leukemia. A large number of the aberrant regulation mechanisms in the Wnt pathway that lead to maintenance, progress, and relapse of chronic leukemia have been revealed. Furthermore, in the recent years, there have been made promising experimental, preclinical researches, targeting the Wnt pathway, particularly successfully in CLL. Further preclinical studies and first clinical results remain to be seen. 

## Figures and Tables

**Table 1:** Overview of recent studies targeting the Wnt pathway in CLL.

Study	Compound	Target in Wnt pathway	Effect
Lu et al. [30]	Ethacrynic acid	(i) Binding to lef-1 protein (ii) Destabilization the lef-1/*β*-catenin complex	(i) Decrease of the survival of cll cells (ii) Induction of cell death in primary cll cells

Jin et al. [42]	Amides of ethacrynic acid	Modifying of sulfhydryl groups present on transcription factors in the Wnt pathway	Decrease in cll cells survival

Lu et al. [43]	Salinomycin	(i) Depression of lpr6 level (ii) Decrease of expression of Wnt target genes (lef-1, cyclin d1)	Limitation of cll cells survival

Razavi et al. [44]	Nitric oxide-donating acetylsalicylic acid (no-asa)	(i) Cleavage of *β*-catenin (ii) Downregulation of *β*-catenin/lef-1 targets	(i) Induction of apoptosis in cll cells (ii) Tumor inhibition rate of 83.4%

Gandhirajan et al. [45]	Small interfering RNA (siRNA) Cgp049090 Pkf115-584	(i) Antagonization of lef-1 (ii) Disruption of the *β*-catenin/lef-1 interaction	(i) Increase of apoptosis of cll cells (ii) Tumor inhibition of 69% and 57%
